# *Cordyceps sinensis* Increases Hypoxia Tolerance by Inducing Heme Oxygenase-1 and Metallothionein via Nrf2 Activation in Human Lung Epithelial Cells

**DOI:** 10.1155/2013/569206

**Published:** 2013-08-26

**Authors:** Mrinalini Singh, Rajkumar Tulsawani, Praveen Koganti, Amitabh Chauhan, Manimaran Manickam, Kshipra Misra

**Affiliations:** Defence Institute of Physiology and Allied Science, Lucknow Road, Timarpur, Delhi 110 054, India

## Abstract

*Cordyceps sinensis*, an edible mushroom growing in Himalayan regions, is widely recognized in traditional system of medicine. In the present study, we report the efficacy of *Cordyceps sinensis* in facilitating tolerance to hypoxia using A549 cell line as a model system. Treatment with aqueous extract of *Cordyceps sinensis* appreciably attenuated hypoxia induced ROS generation, oxidation of lipids and proteins and maintained antioxidant status similar to that of controls via induction of antioxidant gene HO1 (heme oxygenase-1), MT (metallothionein) and Nrf2 (nuclear factor erythroid-derived 2-like 2). In contrast, lower level of NF**κ**B (nuclear factor kappaB) and tumor necrosis factor-**α** observed which might be due to higher levels of HO1, MT and transforming growth factor-**β**. Further, increase in HIF1 (hypoxia inducible factor-1) and its regulated genes; erythropoietin, vascular endothelial growth factor, and glucose transporter-1 was observed. Interestingly, *Cordyceps sinensis* treatment under normoxia did not regulate the expression HIF1, NF**κ**B and their regulated genes evidencing that *Cordyceps sinensis* per se did not have an effect on these transcription factors. Overall, *Cordyceps sinensis* treatment inhibited hypoxia induced oxidative stress by maintaining higher cellular Nrf2, HIF1 and lowering NF**κ**B levels. These findings provide a basis for possible use of Cordyceps sinensis in tolerating hypoxia.

## 1. Introduction

Acclimatization is a major problem for people travelling to high altitudes for the first time. Adverse environmental conditions such as extreme cold, hypoxia, low humidity, high wind velocity, and high intensity of solar radiation [1–3] result in the risk of altitude sickness worldwide. The common problems are acute mountain sickness (AMS), insomnia, lack of appetite, tiredness, lethargy, upset stomach, disinclination to work, bone and muscle degradation, high-altitude pulmonary edema (HAPE), high-altitude cerebral edema (HACE), and all resulted in decrease in physical and mental performance in unacclimatized individuals [4–8]. These problems may escalate rapidly and the results may also be lethal sometimes.

For an individual or cells to adapt to hypoxic conditions, they must be able to sense changes in oxygen tension and respond accordingly. The initiation of these responses can be rapid involving biochemical homeostasis, reprogramming of transcription factors and gene expression, and these changes lead to the production of proteins that exert a protective effect on the cell. Major oxygen and redox-sensitive transcriptional factors (TFs) are nuclear factor- (erythroid-derived 2-) like 2 (Nrf2), hypoxia-inducible factor 1 (HIF1), and nuclear factor kappa-B (NF*κ*B). Expression of antioxidant-responsive-element- (ARE-) driven genes and enzymes is directed by Nrf2 cap'n'collar bZIP transcription factors. These include glutathione peroxidase (GPx), glutathione-S-transferase (GST), heme oxygenase (HO), superoxide dismutase (SOD), and ferritin. HIF1 is selectively stabilized in hypoxia and this in turn leads to activation of several genes such as erythropoietin (EPO) that promotes erythropoiesis, nitric oxide synthase (NOS) that modulates vascular tone, vascular endothelial growth factor (VEGF) that promotes angiogenesis, and glucose transporter-1 (GLUT1) that regulates energy metabolism. Conversely, NF*κ*B activates genes particularly involved in the inflammatory response, as well as in modulating the cellular response to oxidative injury. NF*κ*B plays an important role in the innate and adaptive immunity and cellular survival through the induction of genetic networks [9, 10]. All these events are essential for the survival of cell to hypoxic environment. 

Herbs have been used throughout history to enhance physical performance, but scientific scrutiny with controlled clinical trials has only recently been used to study such effects. Several herbs are currently used to enhance physical performance regardless of scientific evidence of effect: one potential supplement not yet well-characterized is* Cordyceps sinensis *(Berk.) Sacc, a fungus endemic to Himalayan alpine habitats (at an elevation of 3600–5000 m). Purported effects of the fungus suggested a wide range of biological functions such as use as an aphrodisiac [11], analgesic [12], and immune modulator [13] and free radical scavenger [14]. Human clinical trials have demonstrated the effectiveness of *Cordyceps sinensis* fermented mycelia in combating decreased libido and virility [15, 16]. In a clinical study of elderly patients with chronic fatigue, results indicated that most of the subjects treated with *Cordyceps sinensis* pure mycelium reported a significant clinical improvement in the areas of fatigue, cold intolerance, dizziness, frequent nocturia, tinnitus, hyposexuality, and amnesia, while no improvement was reported in the placebo group [17–19]. Inhabitants in the high altitude mountain regions of Tibet and Nepal consume *Cordyceps sinensis* claiming that it gives them energy and offsets the symptoms of high altitude sickness while in West it is consumed by both athletes and elderly people. In recent years, *Cordyceps sinensis *has been investigated in animal and *in vitro *studies for antiaging effects, activity on sexual function, and immune modulation, among other potential uses [20, 21].

Our study on the protective effect of *Cordyceps sinensis* supplementation on hypoxia-induced oxidative stress in lung epithelium cells (A549) is the first of its kind, wherein the effectiveness of *Cordyceps sinensis* in ameliorating the oxidative stress to hypoxia was studied at the molecular level. Further, evaluation of *Cordyceps sinensis *in acclimatization process in high altitude ailments could also enhance the possibility use *Cordyceps sinensis *as a potent naturaltherapeutic agent. In view of above, the present study was designed to evaluate the molecular mechanisms of action of *Cordyceps sinensis *in the process of hypoxia tolerance.

## 2. Material and Methods

### 2.1. Apparatus

HPLC Waters, ASE 350 Dionex Corporation (Sunnyvale, CA, USA), Spectrophotometer Bio-Rad. ELISA reader (Molecular Devices, USA), Spectrofluorimeter (Varian, USA).

### 2.2. Reagents

1,1′-Diphenyl-2-picrylhydrazly [DPPH], 3,4,5-trihydroxybenzoic acid [Gallic Acid], TPTZ [2,4,6-tripyridy-s-triazine], 6 Hydroxy-2,5,7,8-Tetramethylchroman-2-Carboxylic acid [Trolox], Rutin, Folin-Ciocalteu reagent and all other chemicals were from Sigma Aldrich Chemicals, USA. Specific antibodies against all HIF1, NF*κ*B, Nrf2, TNF*α*, TGF*β*, VEGF, EPO, GLUT1, HO1, MT1 and HRP conjugated secondary antibodies were purchased from Santa Cruz Biotechnology, Inc., California, USA.

### 2.3. Collection of Plant Material

*Cordyceps sinensis* mycelia (Voucher specimen DIP-CS/2011) were obtained from Defence Institute of Bio Energy Research, Haldwani. *Cordyceps sinensis* was collected during the rainy season (May-June) from wood logs and tree stumps from the hilly regions (at an altitude of over 4000 m) of the Northwest Himalayas at different locations in Pithoragarh, Uttarakhand, India, where the plant grows widely under natural conditions. Only the mature fruiting bodies (seen as reddish-brown open caps) were selected, removed, and washed with nanopure water, dried under shade in a clean, dust-free environment, and milled into powder using pestle and mortar.

### 2.4. Extraction Procedure

Water extract of *Cordyceps sinensis *was prepared using Accelerated Solvent Extraction system (ASE 350) equipped with a solvent controller unit (Dionex Corporation Sunnyvale, CA, USA). The extraction was carried out in triplicate using water as a solvent. The extraction procedure was as follows: (i) the powdered sample was loaded into cell, (ii) cell was filled with highly pure grade water up to a pressure of 1500 psi, (iii) static extraction was for 15 min, (iv) cell was rinsed with extraction solvent (60% of cell volume) and the solvent is purged from cell with N2 gas (viii), and finally depressurization takes place. Between extractions, a rinse of the complete system was made in order to overcome any extract carryover. The extract was lyophilized (Allied Frost, India) and stored at 4°C.

### 2.5. HPLC Fingerprinting

HPLC analysis of the water extract of *Cordyceps sinensis* was performed using Waters HPLC system (Waters Corporation, USA) equipped with Waters 515 HPLC pump, Waters 717 plus autosampler, and Waters 2487 UV detector. Separation was performed in a symmetry C18 250 mm × 4.7 mm ID, 5 *μ*m column (Waters, USA) by maintaining the isocratic flow rate (1 mL/min) of the mobile phase (0.01 M KH_2_PO_4 _pH 3.7 : methanol 90 : 10) and peaks were detected at 260 nm absorbance.

### 2.6. Determination of Total Phenol Content

The total phenolic content in extract was estimated using Folin-Ciocalteu reagent (FCR) based assay using 20 *μ*L of stock solution (1 mg/mL) of the extract, 80 *μ*l of water, and 500 *μ*L of FCR [22]. After 5 min incubation in dark at room temperature, 400 *μ*L of 7.5% sodium carbonate solution was added. The mixture was further incubated in dark for 30 min at room temperature and absorbance was read at 765 nm. Total phenols (mg/g) in the extract were expressed as gallic acid equivalent (GAE), using standard curve prepared from gallic acid (0.1 mg/mL) solution.

### 2.7. Determination of Total Flavonoid Content

Total flavonoid content was estimated by aluminum chloride (AlCl_3_) colorimetric assay as described elsewhere [23] using rutin as a standard. 1 mL of extract was added to 4 mL distilled water and subsequently 0.3 mL of 5% NaNO_2_ solution was added. After 5 min, 0.3 mL of 10% AlCl_3_ solution was added and allowed to stand for 5 min, and then 0.2 mL of 4% NaOH solution was added to the mixture and the volume was adjusted up to 10 mL with distilled water. Absorbance of the mixture was read at 510 nm. Total flavonoid content (mg/g) in the extract was expressed as rutin equivalent.

### 2.8. FRAP Assay

The FRAP assay was estimated using method described elsewhere [24]. The fresh FRAP working solution was prepared by mixing 25 mL of 300 mM acetate buffer (3.1 g C_2_H_3_NaO_2 _3H_2_O and 16 mL C_2_H_4_O_2_, pH 3.6), 2.5 mL of 10 mM TPTZ (2,4,6-tripyridyl-s-triazine in 40 mM HCl), and 2.5 mL of 20 mM FeCl_3_·6H_2_O solution which was warmed at 37°C prior to use. 150 *μ*L of extract (1 mg/mL) was allowed to react with 2850 *μ*L of the FRAP solution for 30 min in dark and the absorbance of the color developed was read at 593 nm. Results were expressed as mg Trolox equivalent/g of extract, using standard curve prepared from Trolox solution.

### 2.9. DPPH Assay

The DPPH assay was estimated using method described elsewhere [25]. DPPH assay stock solution was prepared by dissolving 24 mg DPPH with 100 mL methanol and then stored at −20°C until needed. The working solution was obtained by mixing 10 mL stock solution with 45 mL methanol to obtain an absorbance of 1.10 ± 0.02 units at 515 nm using the spectrophotometer. 150 *μ*L of leaf extract solution was allowed to react with 2850 *μ*L of the DPPH solution for 2 h in the dark. Then the absorbance was measured at 515 nm. The standard curve was linear between 25 and 200 ppm Trolox. Results are expressed in mg of TE/g of extract. 

### 2.10. 2′-Azino-bis(3-ethylbenzothiazoline-6-sulfonic Acid) Diammonium Salt (ABTS) Assay

The ABTS assay was performed using method described elsewhere [26]. The stock ABTS solution (7 mM) and potassium persulphate (2.45 mM) was mixed in 1 : 1 ratio and incubated overnight in dark at room temperature. The assay was performed by incubation of 250 *μ*L of chemically generated ABTS^•+^ radical in potassium persulfate with 10 *μ*L of extracts or ascorbic acid standard. Absorbance was measured spectrophotometrically at 734 nm for intervals of 1, 2, 5, 10, and 15 min after addition of extract or standard. The extracts without ABTS^•+^ were used as assay control. 

### 2.11. Cells and Culture Conditions

A549 cells (1 × 10^5^ cells/mL) were plated in tissue culture flasks and incubated in normoxia (21% O_2_, 74% N_2_, and 5% CO_2_) at 37°C in DMEM medium (Sigma, St. Louis, USA) supplemented with 10% heat-inactivated fetal bovine serum (Sigma, St. Louis, USA) and penicillin-streptomycin (50 *μ*g/mL, Invitrogen). The hypoxic conditions were achieved by culturing cells in an incubator (Jouan, Saint-Nazaire, France) with a 0.5% O_2_, 5% CO_2_, and 94% N_2_ atmosphere. Cells were treated with *Cordyceps sinensis* extract (2.5 mg/mL stock) dissolved in culture medium and after an hour of treatment, cells were exposed to hypoxia.

### 2.12. MTT (Methylthiazole Tetrazolium) Cytotoxicity Assay

The MTT assay, which is based on conversion of yellow tetrazolium salt to purple-formazan crystals by metabolically active cells, provides a quantitative determination of viable cells. Cells seeded at the density of 10,000 per well in 96 well tissue culture plates were allowed to adhere for 24 h at 37°C. Cells were then treated with 2.5, 5, 10, 25, 50, 100, 250, 500, and 1000 *μ*g/mL concentrations of *Cordyceps sinensis *dissolved in DMEM media. Cells were exposed to normoxia and hypoxia for 24 h or 48 h and cytotoxicity was assessed by MTT assay. Briefly, 50 *μ*L of MTT (1 mg/mL) was added to each well and incubated for 4 h at 37°C. Formazan crystals were solubilized in 100 *μ*L of DMSO by incubating in shaking condition at room temperature for 5 min. Absorbance was taken at 570 nm with 630 nm as reference filter. Absorbance given by untreated cells was taken as 100% cell survival.

### 2.13. Biochemical Analysis

After normoxic and hypoxic exposure, the cells were harvested by trypsinization and washed with sterile phosphate buffered saline (PBS; pH 7.4). The cells were then sonicated for 10 sec in PBS and centrifuged (Sigma Co., Munich, Germany) at 1500 g for 10 min at 4°C. The pellet containing tissue/cell debris was discarded and the supernatant was used to determine ROS, GSH, lipid peroxidation, the protein oxidation, GR, GPx, and SOD activities. The protein content in the homogenate was determined by the method of Lowry et al. [27]. 

### 2.14. Assay for Intracellular Redox State

Intracellular redox state levels were measured using the fluorescent dye 2,7-dichlorofluorescein diacetate (H2-DCFH-DA). Briefly, cells were washed once with HBSS and incubated in the same buffer containing 5–10 *μ*g of DCFH-DA for 30 min at 37°C. Intracellular fluorescence was detected with excitation at 485 nm and emission at 530 nm using Spectra Max Gemini EM (Molecular Devices, Sunnyvale, CA).

### 2.15. Lipid Peroxidation

Lipid peroxidation was assessed by measuring malondialdehyde (MDA) formed by thiobarbiturate (TBA) reaction as thiobarbituric acid reactive substances (TBARS), using the method described elsewhere [28]. Thiobarbiturate was used as the standard, and the level of lipid peroxides was expressed as nmol MDA/mg protein. 

### 2.16. Protein Oxidation

Protein oxidation was measured by determining the carbonyl groups after derivatization with 2,4-dinitrophenyl hydrazine (DNPH) [29]. Carbonyl content was calculated from its molar absorption coefficient as 22,000 m^−1^ cm^−1^ and results were expressed as nmol protein carbonyl per mg protein. Briefly, equal volumes of supernatant and 10 mM DNPH/2M HCl were incubated for 60 min at 50°C. Protein was then precipitated with 20% trichloroacetic acid (TCA), and unreacted DNPH was removed by centrifugation at 1400 g for 10 min. The precipitate was washed three times with a cold ethanol/ethyl acetate (1 : 1) mixture, and finally the precipitate was dissolved in 1 M NaOH. The absorbance was measured at 450 nm and the carbonyl content was obtained as nmol/mg protein.

### 2.17. Enzymatic and Nonenzymatic Antioxidants

Reduced glutathione (GSH) levels were measured fluorometrically by the method described elsewhere [30]. The activities of glutathione peroxidases (GPx), superoxide dismutase (SOD), and glutathione reductase (GR) were determined using commercial kits (Randox) as per the manufacturer's instructions. 

### 2.18. Cellular Protein Preparation and Western Blotting

Following indicated treatments, cells were washed thrice with ice-cold phosphate buffered saline (PBS) and lysed in ice-cold lysis buffer (50 mM Tris-HCl, pH 7.5, with 120 mM NaCl, 10 mM sodium fluoride, 10 mM sodium pyrophosphate, 2 mM EDTA, 1 mM sodium orthovanadate, 1 mM phenylmethylsulfonyl fluoride, 1% NP-40, and protease inhibitor cocktail). Cellular lysates were clarified by centrifugation at 12,000 rpm for 30 minutes. 40 *μ*g of protein was resolved on 10–12% SDS-polyacrylamide gel which was subsequently transferred onto a nitrocellulose membrane (0.45 *μ*). The membranes were probed with respective primary antibodies (Nrf2, HO1, MT, NF*κ*B, TNF*α*, TGF*β* EPO, GLUT1, VEGF) followed by HRP conjugated secondary antibodies. Immunoblots were detected by chemiluminescence reagent (Sigma, USA) and the bands were developed using X-ray films (Kodak, Rochester, NY, USA). Quantification was performed by densitometry using Labworks software (UVP BioImaging Systems).

### 2.19. Data Analysis

All the experiments were performed on three different occasions and data are presented as mean ± SE. The data was analyzed by one-way ANOVA followed by Dunnet's test for comparing control and the various groups, using GraphPad software. Statistical significance was estimated at the 5% level. 

## 3. Results 

### 3.1. Chemical Standardization of Water Extract of Cordyceps Sinensis by Its Total Phenolics, Flavonoid Content, Antioxidant Activity, and Fingerprinting

**Figure 1:**
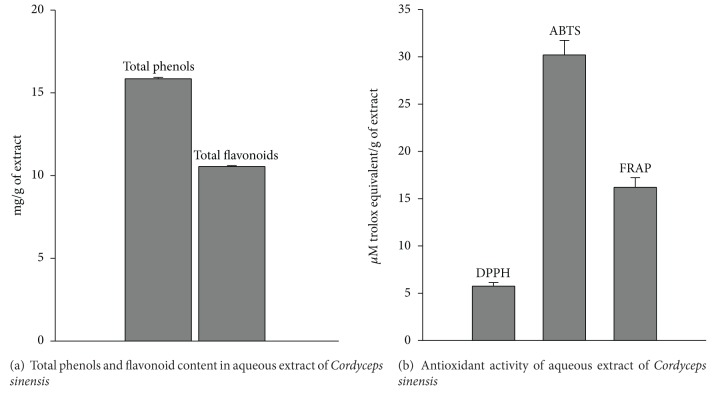
Total phenols, flavonoid content and antioxidant activity of aqueous extract of *Cordyceps sinensis. *

**Figure 2:**
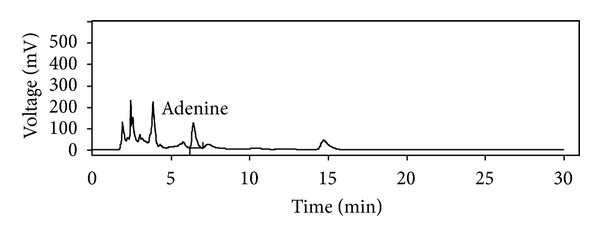
RP-HPLC chromatogram of aqueous extract of *Cordyceps sinensis. *

Figure 1 depicts % yield, total phenolics, flavonoid content, and antioxidant activity of water extract of *Cordyceps sinensis. *The extract showed 15.9 ± 0.08 mg/g of total phenols and 10.6 ± 0.05 mg/g of flavonoid content (Figure 1(a)). The antioxidant activity of extract was found to be 5.75 ± 0.38, 30.2 ± 1.54, 16.2 ± 1.02 *μ*M TE/g of extract as evident by DPPH, ABTS, and FRAP assays, respectively (Figure 1(b)).  Figure 2 shows HPLC chromatograms at 260 nm. The presence of adenine, the marker compound of *Cordyceps sinensis*, was evident in the extract with a retention time of 6.43. Adenine was found to be 0.71% w/w. 

### 3.2. Effect of Cordyceps sinensis on Cell Viability and ROS Production in Hypoxia Exposed A549 Cells

**Figure 3:**
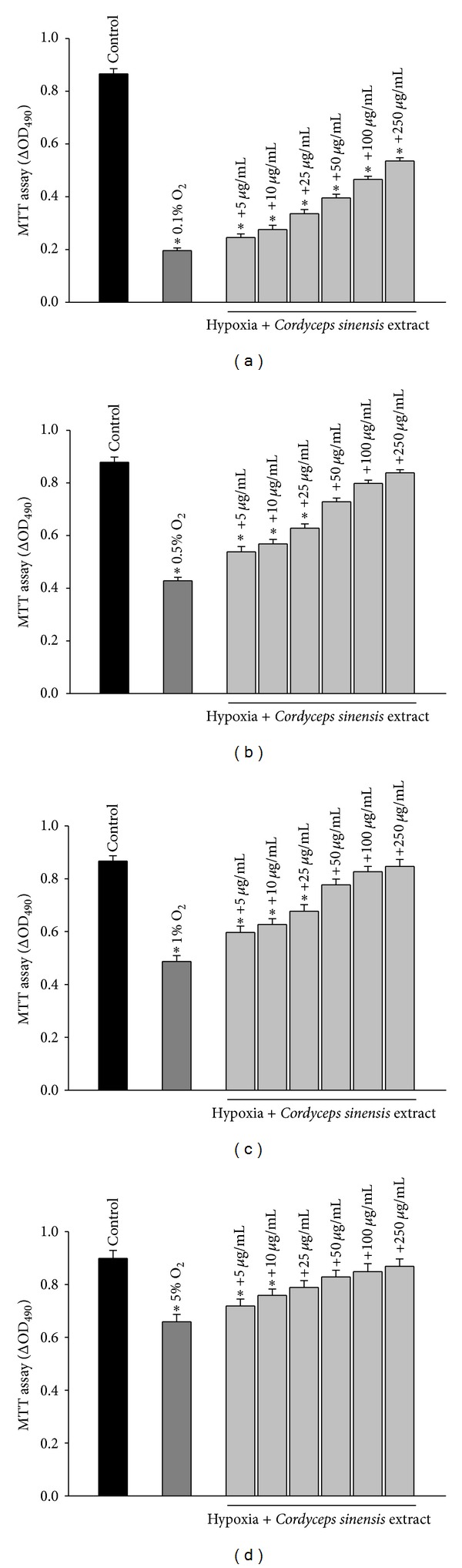
Cytoprotective effects of aqueous extract of *Cordyceps sinensis *in A549 cells exposed to different oxygen percentages (0.1, 0.5%, 1%, or 5% O_2_; (a)–(d) for 48 h. Cells exposed to hypoxia showed cell death (dark gray bar) in comparison to unexposed cells (black bar) which was dependent on oxygen percentage. Treatment of cells with aqueous extract of *Cordyceps sinensis* increased cell survival following hypoxia exposure and the efficacy of extract was dose dependent (light gray bars). *P < 0.05.

**Figure 4:**
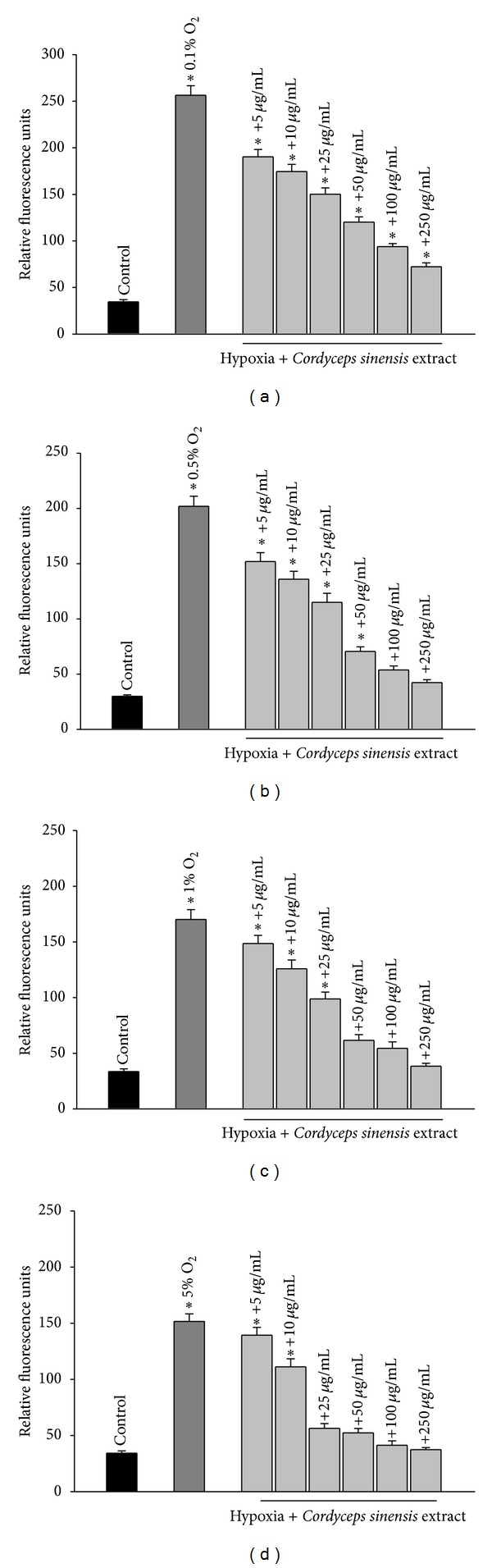
Antioxidative effects of aqueous extract of *Cordyceps sinensis in* A549 cells exposed to different oxygen percentages (0.1, 0.5%, 1%, or 5% O_2_; (a)–(d)) for 48 h. Cells exposed to hypoxia showed elevated levels of reactive oxygen species (dark gray bars) compared to unexposed cells (black bars) which was dependent on oxygen percentage. *Cordyceps sinensis *decreased reactive oxygen species following hypoxia exposure and the efficacy of extract was dose dependent (light gray bars). *P < 0.05.

We determined the effect of hypoxia alone using different percentages of oxygen (0.1%, 0.5%, 1%, and 5% O_2_) or in combination with various concentrations of *Cordyceps sinensis* extract (5–250 *μ*g/mL) on cell viability and generation of reactive oxygen species. Cells exposed to varying percentage of oxygen showed increased reactive oxygen species and cell death. The cytotoxic effect of hypoxia was dependent on the concentration of oxygen (Figures 3(a)–3(d) and 4(a)–4(d), dark gray bars). The treatment of cells with various concentrations of extract prevented cell death and generation of reactive oxygen species following hypoxia exposure and the protective effects were dose dependent (Figures 3(a)–3(d) and 4(a)–4(d), light gray bars). Because 0.5% O_2_ showed approximately 51% cell death and 250 *μ*g/mL of *Cordyceps sinensis* extract showed maximal effects on cell viability and ROS generation, all subsequent experiments were conducted using the above-mentioned percentage of oxygen and concentration of extract. 

### 3.3. Lipid Peroxidation

**Figure 5:**
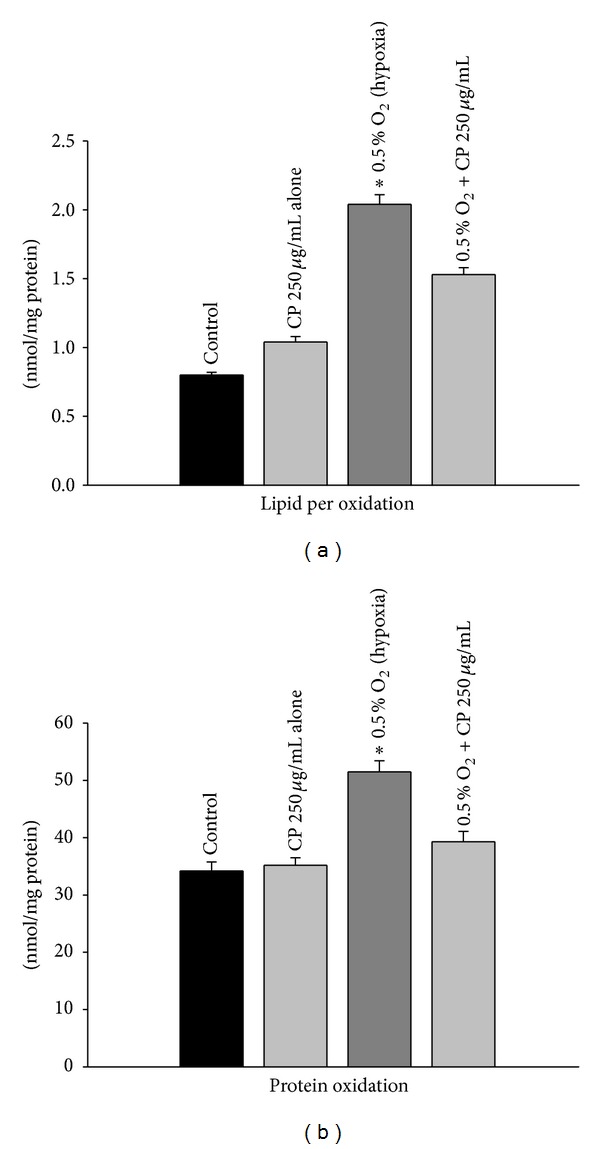
Lipid peroxidation (TBARS, thiobarbituric acid reactive substance) and protein oxidation (carbonyl groups after derivatization of proteins with dinitrophenylhydrazine) in A549 cells were determined after (0.5% O_2_) hypoxia exposure for 48 h by spectrophotometric measurement. A significant increase in both lipid peroxidation and protein oxidation was observed in cells exposed to hypoxia and such changes were attenuated in hypoxic cells treated with *Cordyceps sinensis *extract. Experiments were done in triplicate. Values are mean ± SE of three individual experiments. *P < 0.05.

Since hypoxia exposure led to increased oxidative stress, we determined the levels of lipid peroxidation. Exposure to hypoxia caused a marked increase in lipid peroxidation as observed by an increase in MDA levels. However, *Cordyceps sinensis *treatment significantly inhibited the hypoxia-induced lipid peroxidation as compared with the respective hypoxia exposed cells (Figure 5(a); *P* < 0.05). No significant difference was observed in the normoxic cells and *Cordyceps sinensis* treated cells kept in normoxic conditions.

### 3.4. Protein Oxidation

The effect of hypoxia on oxidation of proteins was measured by determining protein carbonyl contents in A549 cells after derivatization with DNPH. The results showed a considerable increase in protein oxidation in cells exposed to hypoxia as compared to the control. *Cordyceps sinensis *treatment appreciably inhibited the formation of protein carbonyls levels (Figure 5(b); *P* < 0.05) after exposure to hypoxia. No significant change was observed in protein oxidation in cells treated with* Cordyceps sinensis* kept in normoxic conditions relative to normoxic control cells.

### 3.5. Glutathione Status and Antioxidant Enzyme Activity

To assess the level of endogenous nonenzymatic antioxidant, GSH was determined. Exposure of A549 cells to hypobaric hypoxia resulted in a significant decrease in GSH levels as compared with normoxic control cells (Table 1; *P* < 0.05). *Cordyceps sinensis *treatment significantly increased GSH levels after exposure to hypoxia though no difference was observed between *Cordyceps sinensis *treatment normoxic cells and control normoxic cells (Table 1).

As shown in Table 1, exposure to hypoxia resulted in a significant increase in SOD activity and a decrease in GPx and GR activities relative to normoxic cells (*P* < 0.05). *Cordyceps sinensis *treatment maintained these enzymes levels similar to the control values, indicating reduced oxidative stress.

### 3.6. Heme Oxygenase-1 and Metallothionein Levels

**Figure 6:**
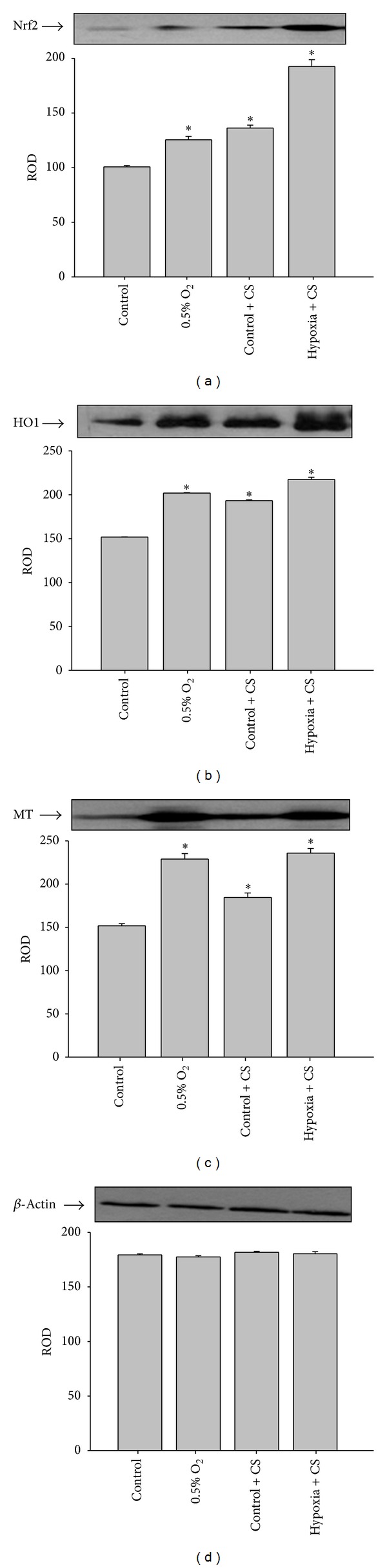
Effect of *Cordyceps sinensis *treatment on the expression of antioxidant transcription factor (Nrf2; (a)) and its regulated genes heme oxygenase-1 (HO1; (b)) and metallothionein 1 (MT; (c)) in A549 cells (0.5% O_2_) hypoxia exposure for 48 h. There was a significant increase in Nrf2, HO1, and MT levels following hypoxia exposure and also in the presence of *Cordyceps sinensis *extract. The figures are representative of three independent experiments. These were normalized with actin to observe any change in expression.

Considering that *Cordyceps sinensis* treatment has significantly inhibited ROS and maintained antioxidant status of hypoxia exposed cells, we sought whether this ability to prevent oxidative stress is mediated via Nrf2, HO1 and MT. Therefore, the relative levels of Nrf2, HO1 and MT were determined by immunoblotting. Exposure of cells to hypoxia resulted in a significant increase in Nrf2, HO1, and MT relative to normoxic control cells (Figure 6).* Cordyceps sinensis* treatment and hypoxia exposure further increased expression of Nrf2, HO1, and MT levels compared to normoxic cells.

### 3.7. Role of NF*κ*B in Cellular Protection

**Figure 7:**
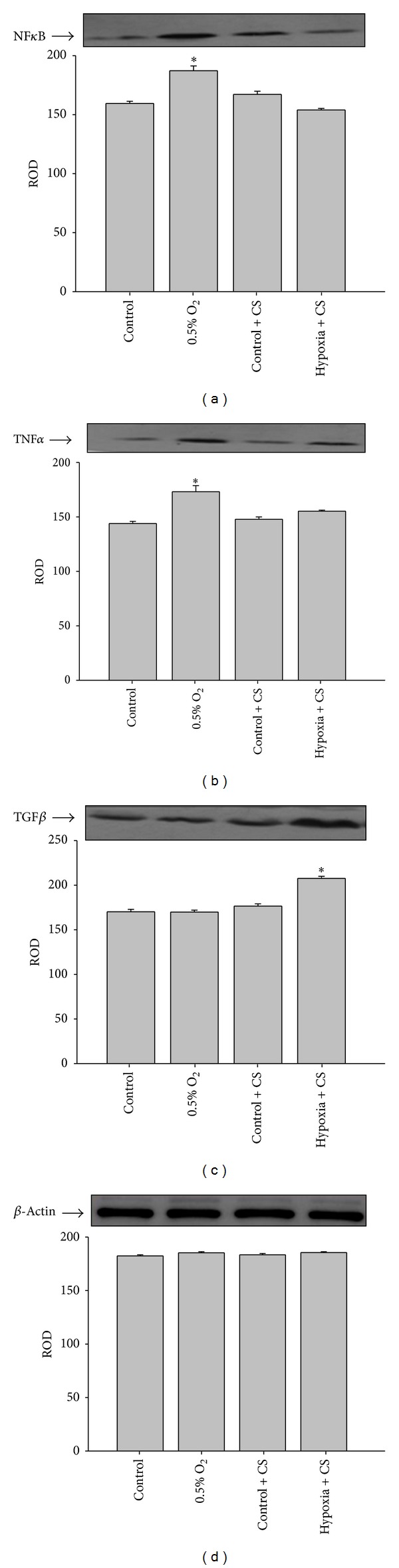
Western blot analyses showed significant increase in NF*κ*B (a) and pro-inflammatory cytokines TNF*α* (b) while no change was observed in levels of anti-inflammatory cytokines TGF*β* (c) in A549 cells following hypoxia (0.5% O_2_) exposure for 48 h. The treatment of cells with extract prevented increase in NF*κ*B and TNF*α* levels following hypoxia exposure. Further, an increase in TGF*β* levels was also evident in *Cordyceps sinensis *treated cells. The figures are representative of three independent experiments. These were normalized with *β*-actin (d) to observe any change in expression.

To investigate the role of NF*κ*B-mediated mechanisms in the induction of protection, the effect of *Cordyceps sinensis* treatment on NF*κ*B and its target genes were examined. It is a key transcriptional factor that regulates inflammatory mediators. We determined the relative levels of active NF*κ*B by immunoblotting. Exposure of cells to hypoxia resulted in a marked increase in NF*κ*B levels; however, *Cordyceps sinensis* treatment significantly attenuated NF*κ*B expression (Figure 7). 

Since NF*κ*B regulates inflammatory mediators, we determined pro- and anti-inflammatory molecules like TNF*α* and TGF*β*. There was a considerable increase in TNF*α* levels upon hypoxic exposure relative to control cells. However, no change in TGF*β* levels was noticed in the cells exposed to hypoxia. *Cordyceps sinensis* treatment significantly attenuated hypoxia induced increase in TNF*α* level. Interestingly, the levels of TGF*β* increased upon *Cordyceps sinensis* treatment (Figure 7).

### 3.8. Effect of Cordyceps sinensis on HIF1 Expression and Its Regulated Genes

**Figure 8:**
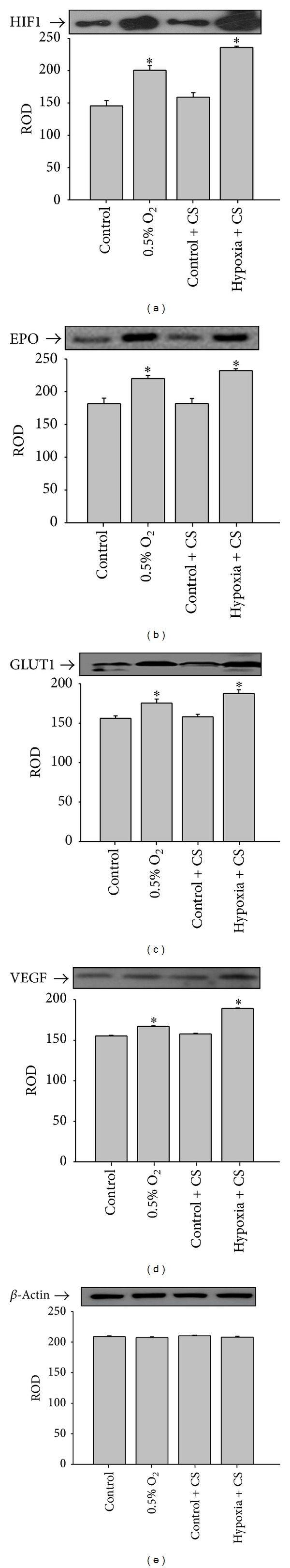
Immunoblot analysis of HIF1 (a) and HIF1 regulated genes, namely, EPO (b), GLUT1 (c), and VEGF (d) in A549 cells following hypoxia (0.5 % O_2_) exposure for 48 h. The treatment of cells with *Cordyceps sinensis *increased levels of HIF1, EPO, GLUT1, and VEGF following hypoxia exposure. The figures are representative of three independent experiments. These were normalized with *β*-actin (e) to observe any change in expression.

HIF1 level was measured in A549 cells by immunoblotting. The results showed a marked increase in HIF1 expression during exposure to hypoxia. Interestingly, *Cordyceps sinensis* treated cells under hypoxic condition showed further increase in HIF1 protein (Figure 8). To know whether increased HIF1 levels in hypoxia and *Cordyceps sinensis* treated cells resulted in increased expression of its downregulated genes, western blotting was performed for EPO, GLUT1, and VEGF, which promote erythropoiesis, glucose transport, and angiogenesis, respectively. A significant increase in VEGF and GLUT1 protein levels was observed in hypoxia and *Cordyceps sinensis* treated cells. EPO protein increased slightly following hypoxia and *Cordyceps sinensis* treatment (Figure 8).

## 4. Discussion

In the preliminary experiments, A549 cells were exposed to different percentages of oxygen 0.1%, 0.5%, 1%, and 5% for 24–48 h duration and the results showed 70–77%, 42–51%, 32–44%, and 15–27% cell death with an increase of 6.6–7.4, 5.3–6.7, 4.3–5.1, and 3.5–4.3 fold in reactive oxygen species, respectively. The findings suggested that the cell death was due to increased oxidative stress governed by percentage of oxygen and duration of hypoxia exposure. Based on preliminary data optimal percentage of oxygen was found to be 0.5% O_2_ as 0.1% O_2_ percentage of oxygen reduced cell survival to 23–30% while 1% and 5% O_2_ resulted in minimal cell death. In a separate study, cells were exposed to various concentrations of *Cordyceps sinensis* extract (2.5–1000 *μ*g/mL) for 24 h and 48 h, and A549 cells did not show any toxicity *per se*. The concentration of *Cordyceps sinensis* extract below 5 *μ*g/mL was not effective and cells treated above 250 *μ*g/mL could not provide additional improvement in efficacy of extract; hence, data of effective dose range 5–250 *μ*g/mL is only presented in Figures 3(a)–3(d) and 4(a)–4(d). The effective dose range for aqueous extract of *Cordyceps sinensis* under hypoxia exposure of 48 h was found to be 5–250 *μ*g/mL; however, the best results were obtained with 250 *μ*g/mL against all the percentage of oxygen. Therefore, we have used 0.5% O_2_ and 250 *μ*g/mL of *Cordyceps sinensis* extract in all our studies except MTT and ROS. 

The present study shows that treatment of *Cordyceps sinensis* significantly improves tolerance to hypoxia as revealed by increased Nrf2 and HIF1 and decrease in NF*κ*B transcription factors. This in turn led to increased antioxidant genes, antioxidant enzymes, EPO, VEGF, and GLUT1 levels, facilitating better oxygenation, oxygen delivery, and glucose transport. At the same time decrease in NF*κ*B levels resulted in lower expression of proinflammatory cytokines like TNF*α* and increased expression of anti-inflammatory cytokine TGF*β*. All these changes contributed to the increase in hypoxic tolerance of *Cordyceps sinensis* treated cells. To the best of our knowledge, we have demonstrated for the first time the potency of *Cordyceps sinensis* treatment in inducing tolerance to hypoxia. 

During hypoxia, less oxygen is available to be reduced to H_2_O at cytochrome oxidase, causing accumulation of highly reactive oxygen intermediates (ROIs). Overproduction of ROIs can readily cause oxidative damage to various biological macromolecules resulting in DNA damage, oxidation of key amino acid side chains, formation of protein-protein cross-links, oxidation of the polypeptide backbone resulting in protein fragmentation, and lipid peroxidation. The present study also reports that exposure of A549 cells to hypoxia resulted in an appreciable increase in ROS levels which in turn could be responsible for the observed increase in oxidation of cellular protein and lipids. For hypoxia-associated free radicals, antioxidant can safely interact with free radicals and terminate the chain reaction before vital biological macromolecules are damaged. Cellular antioxidants defense network comprising the well-known endogenous enzymes superoxide dismutase (SOD), catalase (CAT), and glutathione peroxidase (GPx), as well as nonenzymatic antioxidants reduced glutathione (GSH). These participate in maintaining the proper balance of free radicals and antioxidants in the healthy cells. The cells exposed to hypoxia showed a decrease in GSH levels and to cope up with this hypoxia-induced stress, a marked increase in activities of cellular antioxidant enzymes such as SOD, GPx, and GR were observed. However, *Cordyceps sinensis* supplementation maintained the antioxidant enzymes levels similar to those of control values and restores the GSH level. The findings suggested that *Cordyceps sinensis* exerted its robust antioxidant defense abilities by activating endogenous antioxidant enzymes as well as inhibiting protein and lipid peroxidation due to presence of higher amount of phenols, flavonoids, nucleosides (adenosine), nucleobases (adenine and uracil), and polysaccharides; these compounds absorb and neutralize free radicals, quench singlet and triplet oxygen, or decompose peroxides [31]. This result is in agreement with other reports that aqueous extracts of natural and cultured *Cordyceps *species are more effective in free radical scavenging than in hydrophobic systems [32]. Yao's group [33] has reported that the water extracts of *Cordyceps sinensis* mycelia have direct and moderate-to-potent antioxidant activities involving the scavenging of superoxide anion radical, hydroxyl radical, and inhibition of lipid peroxidation. They also proposed that the antioxidant activities of the water extracts may be caused by a combined effect of proteins, polysaccharides, and mannitol or some other compounds in *Cordyceps sinensis* mycelia. However, Li et al. [34] have observed that the water extract of *Cordyceps sinensis* mycelia possessed a strong antioxidant activity and that partially purified polysaccharides from the water extract showed greater antioxidant activities (10-fold to 30-fold) than before purification. Therefore, polysaccharides may be regarded as the key components of the antioxidant activity of *Cordyceps sinensis* mycelia. 

Growing evidence has indicated that cellular redox plays an essential role not only in cell survival but also in cellular signaling pathways such as NF*κ*B. Several laboratories have demonstrated that treatment of cells with H_2_O_2_ (ROIs) can activate the NF*κ*B pathway as reviewed by Bauerle and Henkel [35]. Shih et al. [36] demonstrated that the hydroxyl radical was primarily responsible for activation of NF*κ*B in Jurkat cells and macrophages. During hypoxia elevated levels of ROIs prompted speculation that ROIs may function as common mediators of NF*κ*B activation also. Since NF*κ*B is involved in inflammation, we therefore analyzed the expression of NF*κ*B and its proinflammatory mediator TNF*α* in cells exposed to hypoxia. A marked increase in NF*κ*B and TNF*α* levels was seen during hypoxia; however, *Cordyceps sinensis* treatment + hypoxia markedly inhibited NF*κ*B and TNF*α* expression possibly due to reduction in hypoxia-induced oxidative stress indicating towards cells defense against the hypoxic stress. This hypothesis is in agreement with an earlier report in which evidence showing that nearly all pathways leading to NF*κ*B activation could be blocked by a variety of antioxidants, including pyrrolidine dithiocarbamate (PDTC), N-acetyl-L-cysteine (NAC), glutathione (GSH), or thioredoxin (Txn), or by overexpression of antioxidant enzymes, including superoxide dismutase, glutathione peroxidase, or thioredoxin peroxidase. As aforementioned, *Cordyceps sinensis *possesses robust antioxidant properties. 

Based on the results mentioned above and reported facts, we hypothesized that *Cordyceps sinensis* treatment inhibited oxidative stress and inflammation in the hypoxia exposed cells. Thus, we explored the probability of alterations in the expression of Nrf2 transcription factor, a master regulator of endogenous antioxidative systems [36–38] being involved in cell survival. We found that *Cordyceps sinensis* + hypoxia treatment resulted in a significant increase in Nrf2 levels. Having confirmed the expression of Nrf2, we investigated the levels of HO1 which is known to possess antioxidant and anti-inflammatory activity and regulated by Nrf2 [39]. Our results demonstrated that *Cordyceps sinensis* + hypoxia resulted in higher levels of HO1. We also measured other anti-inflammatory mediators such as TGF*β* and MT levels in A549 cells. The study revealed significant upregulation of TGF*β* and MT after *Cordyceps sinensis* + hypoxia treatment, and all these genes might be responsible for the observed fall in proinflammatory cytokines after exposure to hypoxia. Our results are in accordance with previous report [40] stating that activation of Nrf2 effectively reduces oxidative stress and inflammation in rats. Similarly overexpression of TGF*β*, HO1, and MT facilitates acclimatization and maintains the redox balance in the cells [41, 42]. The capacity of *Cordyceps sinensis* in limiting the oxidative stress is partly mediated by Nrf2 pathway. 

**Figure 9:**
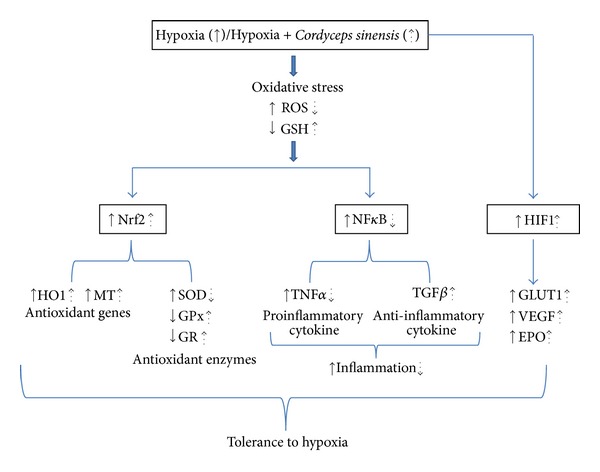
Schematic representations of effects of hypoxia and aqueous extract of *Cordyceps sinensis *in A549 cells. Putative corelation among various transcription factors and mechanism of tolerance to hypoxia, upregulation/downregulation after hypoxia (solid arrow), and upregulation/downregulation after *Cordyceps sinensis *treatment (dotted arrow).

Hypoxia has been shown to activate HIF1, thus an attempt was made to investigate changes in the expression and to define a functional involvement of HIF1, in response to *Cordyceps sinensis* treatment. We found that *Cordyceps sinensis* treatment 1 h before hypoxia exposure resulted in a significant increase in HIF1 levels; this suggests that HIF1-mediated gene expression may be involved in hypoxia-induced tolerance. Therefore, we determined the relative levels of HIF1 regulated genes, EPO, GLUT1, and VEGF by immunoblotting in control and *Cordyceps sinensis* treated cells. *Cordyceps sinensis* treatment under hypoxia resulted in higher EPO levels, increased EPO levels, and increased O_2_ carrying capacity. Further the protein levels of GLUT1 mediating the transport of glucose were increased in hypoxia and *Cordyceps sinensis* + hypoxia cells compared to the control cells, indicating enhanced glucose uptake for continued energy generation in hypoxic environments. Our results are in accordance with previous studies suggest that CordyMax CS-4 supplementation effectively lowers blood glucose and plasma insulin and improves glucose metabolism by enhancing insulin sensitivity [43]. It is presumed that when cells in the tissue are deprived of oxygen, they release angiogenic factor that induce new capillary growth and hence better oxygen transport [6]. Of the known angiogenic factors FGF and VEGF are most commonly expressed [8]. In our study too, we found a significant increase in VEGF protein levels in hypoxic cells and *Cordyceps sinensis* + hypoxia exposed cells relative to control cells. These results indicate that the induction of specific HIF1 and its regulated genes in *Cordyceps sinensis* treated cells following hypoxia is an attempt by cells to initiate the process of acclimatization to escape death. Putative corelation among various transcription factors and mechanism of tolerance offered by aqueous extract of *Cordyceps sinensis* to hypoxia is schematically represented in Figure 9.

To conclude exposure of cells to hypoxia resulted in a significant increase in oxidative stress; further, there was a marked increase in NF*κ*B activity and inflammatory mediators TNF*α*. Treatment of cells by *Cordyceps sinensis* (250 *μ*g/mL) significantly attenuated the oxidative stress in hypoxia. This was attributed to activation of Nrf2-ARE pathway, reduced NF*κ*B activity, and increased oxygen availability via HIF1 signaling mechanisms. Further, higher levels of antioxidant, anti-inflammatory mediators, namely, TGF*β*, HO1, and MT levels in *Cordyceps sinensis* treated cells might also be responsible for tolerance of cells to hypoxia. The findings of the study will help in the development of novel therapeutic strategies to use *Cordyceps sinensis* as a nutraceutical for promoting tolerance to high altitude.

## Figures and Tables

**Table 1:** Protective effects of aqueous extract of *Cordyceps sinensis* on antioxidant status of cells exposed to hypoxia.

Treatment	GPx (U/L)	SOD (U/mL)	GR (U/L)	GSH (mM)
Control	400.7 ± 38.8	24.4 ± 1.74	42.3 ± 2.23	9.01 ± 0.32
Hypoxia	328.0 ± 22.2*	48.4 ± 3.46*	14.9 ± 0.82*	3.34 ± 0.26*
Control + extract	400.9 ± 18.9	26.6 ± 2.02	43.8 ± 2.84	8.83 ± 0.33
Hypoxia + extract	386.9 ± 20.4	32.7 ± 2.46*	44.7 ± 2.62	7.86 ± 0.38

A549 cells were exposed to hypoxia (0.5% O_2_) for 48 h in the absence or presence of aqueous extract of *Cordyceps sinensis* (250 *μ*g/mL). A significant decrease in GSH, GPx, and GR levels observed after hypoxia was restored in *Cordyceps sinensis* treated cells. Further, an increase in SOD activity was observed in cells exposed to hypoxia and the effect was nearly neutralized by *Cordyceps sinensis* treatment. Results are means ± SE of three individual experiments. *P < 0.05.
